# Extracorporeal membrane oxygenation support in post-traumatic cardiopulmonary failure: A 10-year single institutional experience

**DOI:** 10.1097/MD.0000000000006067

**Published:** 2017-02-10

**Authors:** Chun-Yu Lin, Feng-Chun Tsai, Hsiu-An Lee, Yuan-His Tseng

**Affiliations:** a Department of Cardiothoracic and Vascular Surgery, Chang Gung Memorial Hospital, Linkou; b Department of Cardiothoracic and Vascular Surgery, Chang Gung Memorial Hospital, Chiayi, Taiwan.

**Keywords:** ECMO, trauma

## Abstract

Patients with multiple traumas associated with cardiopulmonary failure have a high mortality rate; however, such patients can be temporarily stabilized using extracorporeal membrane oxygenation (ECMO), providing a bridge to rescue therapy. Using a retrospective study design, we aimed to clarify the prognostic factors of post-traumatic ECMO support.

From March 2006 to July 2016, 43 adult patients (mean age, 37.3 ± 15.2 years; 7 females [16.3%]) underwent ECMO because of post-traumatic cardiopulmonary failure. Pre-ECMO demographics, peri-ECMO events, and post-ECMO recoveries were compared between survivors and nonsurvivors.

The most common traumatic insult was traffic collision (n = 30, 69.8%), and involved injury areas included the chest (n = 33, 76.7%), head (n = 14, 32.6%), abdomen (n = 21, 48.8%), and fractures (n = 21, 48.8%). Fifteen patients (34.9%) underwent cardiopulmonary resuscitation and 22 (51.2%) received rescue interventions before ECMO deployment. The mean time interval between trauma and ECMO was 90.6 ± 130.1 hours, and the mode of support was venovenous in 26 patients (60.5%). A total of 26 patients (60.5%) were weaned off of ECMO and 22 (51.6%) survived to discharge, with an overall mean support time of 162.9 ± 182.7 hours. A multivariate regression analysis identified 2 significant predictors for in-hospital mortality: an injury severity score (ISS) >30 (odds ratio [OR], 9.48; 95% confidence interval [CI], 1.04–18.47; *P* = 0.042), and the requirement of renal replacement therapy (RRT) during ECMO (OR, 8.64; 95% CI, 1.73–26.09; *P* = 0.020). These two factors were also significant for the 1-year survival (ISS >30: 12.5%; ISS ≤30, 48.1%, *P* = 0.001) (RRT required, 15.0%; RRT not required, 52.2%, *P* = 0.006).

Using ECMO in selected traumatized patients with cardiopulmonary failure can be a salvage therapy. Prompt intervention before shock-impaired systemic organ perfusion and acute renal failure, especially in high ISS patients, is crucial for both hospital and one-year survival.

## 1 Introduction

Severe trauma can cause a lethal outcome (28–83%) when concomitant pulmonary and/or cardiopulmonary failure occurs.^[1,2]^ Catastrophic hemorrhage with solid organ involvement and acute lung injury following fluid resuscitation may further compromise cardiopulmonary function.^[3–6]^ Extracorporeal membrane oxygenation (ECMO) can effectively stabilize the patients’ hemodynamic status and provide a bridge to rescue therapy.^[7–10]^ This study aimed to clarify the prognostic factors of post-traumatic ECMO support.

## 2 Patients and methods

### 2.1 Patient enrollment and pre-ECMO management

The present study was conducted with approval of the Institutional Ethics Committee (No.2016–0147–5B0). The need for informed consent was waived due to the retrospective nature of the study. All patients with post-traumatic cardiopulmonary failure between March 2006 and July 2016, were evaluated according to the algorithm selection protocol described in our previous reports.^[7,8]^ During the study period, a total of 43 adults underwent ECMO due to post-traumatic pulmonary and/or cardiopulmonary failure.

During pre-ECMO management, active bleeding sources were controlled, fluid resuscitations were maximized, and correctable pulmonary insults were identified and treated before considering ECMO deployment. Patients presenting with refractory shock or experiencing cardiopulmonary resuscitation (CPR) were supported using the venoarterial (VA) mode of ECMO. The venovenous (VV) mode of ECMO was reserved for patients with persistent hypoxia or hypercapnia meeting the Berlin definition of acute respiratory distress syndrome (ARDS),^[11]^ but without contraindicated severe pulmonary hypertension (mean pulmonary artery pressure >45 mmHg), high doses of inotropic support, or a history of cardiac arrest or resuscitation. Patients were enrolled for fast-entry if they were failing to maintain acceptable oxygenation (PaO2/FiO2 <60 at a positive end expiratory pressure [PEEP] >10 mmHg) after 2 hours of aggressive therapy. Some patients initially excluded from fast-entry were placed on ECMO support after exhibiting progressive deterioration and meeting slow-entry criteria.^[12]^

### 2.2 ECMO therapy

Informed consent was obtained from the closest family member before deploying ECMO. The ECMO system (CAPIXO EBS, Emergency Bypass System, Terumo, Tokyo, Japan) consists of a membrane-type oxygenator with a microporous hollow fiber, a CAPIOX SP centrifugal pump employing a straight-path design, and a blood circuit. All components are fully integrated and preassembled to enable rapid and easy setup during emergent situations. Heparin-coated cannula (Medtronic, Bio-Medicus, USA) ensured superior biocompatibility and could be heparin-free in the first 12 hours of deployment if bleeding was a concern. To prevent distal limb ischemia, one 8 Fr. distal perfusion catheter (ARROW, Super Arrow-Flex, USA) was routinely placed on the superficial femoral artery for patients receiving VA ECMO installation with the cut-down technique. The percutaneous Seldinger approach was considered the most expeditious means of vascular access in VV mode. The largest accommodating cannula was positioned to facilitate a high blood flow and full oxygenation support. The circuit pathway most commonly used to minimize recirculation involved blood drainage from the femoral vein and return through the internal jugular vein.

### 2.3 Peri-ECMO care and interventions

The ECMO pump flow was initially maximized to ensure adequate circulatory support and oxygenation. Blood gases, potassium, and ionized calcium concentrations were corrected to prevent unexplained arrhythmia and systemic hypotension. The platelet count was maintained >80,000 cells/mm^3^, and fibrinogen was maintained above 100 mg/dl to reduce the risk of bleeding. If hemorrhage was a concern after ECMO was deployed, anti-fibronlytics were not used. Instead, heparin was held for 12–24 hours, ACT and APTT levels were closely monitored, and the ECMO circuit was changed early, once thrombus was detected. The decision to start heparinization to prevent systemic emboli was based on the individual patient's status, without common consensus or protocol agreement. In general, after emergent surgeries 8 hours without active bleeding or consumption coagulopathy solved by laboratory test, systemic heparinization will be restarted. Early renal replacement therapy (RRT) was aggressively applied if acute renal failure developed during ECMO support. The associated systemic organ injuries were rechecked thoroughly after ECMO stabilized the hemodynamics. Further treatments including survey images, endovascular intervention, or surgical exploration during ECMO support were performed without hesitation whenever indicated. Tracheostomy was performed whenever the patient could be weaned from ECMO, but prolonged intubation was expected.

After clinical stabilization or undergoing hemostatic procedures, all patients were transferred to a specialized trauma intensive care unit for further treatment and observation.

### 2.4 Data collection and statistical analysis

Demographics, traumatic mechanisms, pre-ECMO interventions, laboratory data, ventilator parameters, ECMO therapy, combined surgical procedures, and clinical outcomes were compared between survivors and nonsurvivors. The injury severity score (ISS) and sequential organ failure assessment (SOFA) score were used to evaluate the trauma severity.^[13–15]^ In addition, the serum lactate level, which was routinely checked within 8 hours after ECMO installation, was evaluated. Among patients receiving VA ECMO, survival after VA ECMO (SAVE) score was used to estimate hospital survival.^[16]^ To evaluate the cerebral performance among survived patients, the cerebral performance categories (CPC) scale is analyzed individually on discharge and 1 year after.^[17]^

Statistical analyses were performed using SPSS for Windows (Version 22.0, SPSS Inc, Chicago, IL). Data are presented as means ± standard deviation for continuous variables, and as percentages for categorical data. For all analyses, statistical significance was set at *P* < 0.05. Univariate analyses were performed using independent *t* test, Mann–Whitney *U* test, *χ*^2^ test, or Fisher exact test to determine group differences in clinical demographics, ECMO information, and post-ECMO-associated complications. Significant variables in univariate analyses of in-hospital mortality (*P* < 0.05) were dichotomized based on cutoff values, which were determined in receiver-operating characteristic (ROC) curve analyses. These dichotomized risk factors were tested in a prediction model of in-hospital mortality using a multivariate logistic regression analysis, Hosmer-Lemeshow test, and area under ROC curve (AUROC) analysis.^[18]^ The Kaplan-Meier method was used to construct 1-year cumulative survival curves, which were compared using the log-rank test.

## 3 Results

### 3.1 Patient characteristics and trauma-related profiles

The clinical demographics, trauma mechanisms, involved area, severity, and ventilation parameters for the survivor and mortality groups are provided in Table 1. In both groups, traffic collision was the most prevalent trauma mechanism, accounting for >60% of cases, followed by falling down. Most associated injuries involved the chest area (76.7%), followed by the abdomen (48.8%). A total of 34.9% of the patients underwent cardiopulmonary resuscitation (CPR) and 51.2% underwent salvage interventions before ECMO deployment. The mean time interval from injury to ECMO was 90.6 ± 130.1 hours. Patients with hospital mortality had significantly higher injury scores (ISS, 23.4 ± 9.2 vs. 35.3 ± 11.4, *P* = 0.001; SOFA, 8.1 ± 2.9 vs. 13.9 ± 4.7, *P* = 0.010), greater occurrence of shock (22.7% vs. 57.4%, *P* = 0.044), and greater occurrence of pelvic fractures (14.5% vs. 33.3%, *P* = 0.045) compared to those for patients without hospital mortality.

**Table 1:**
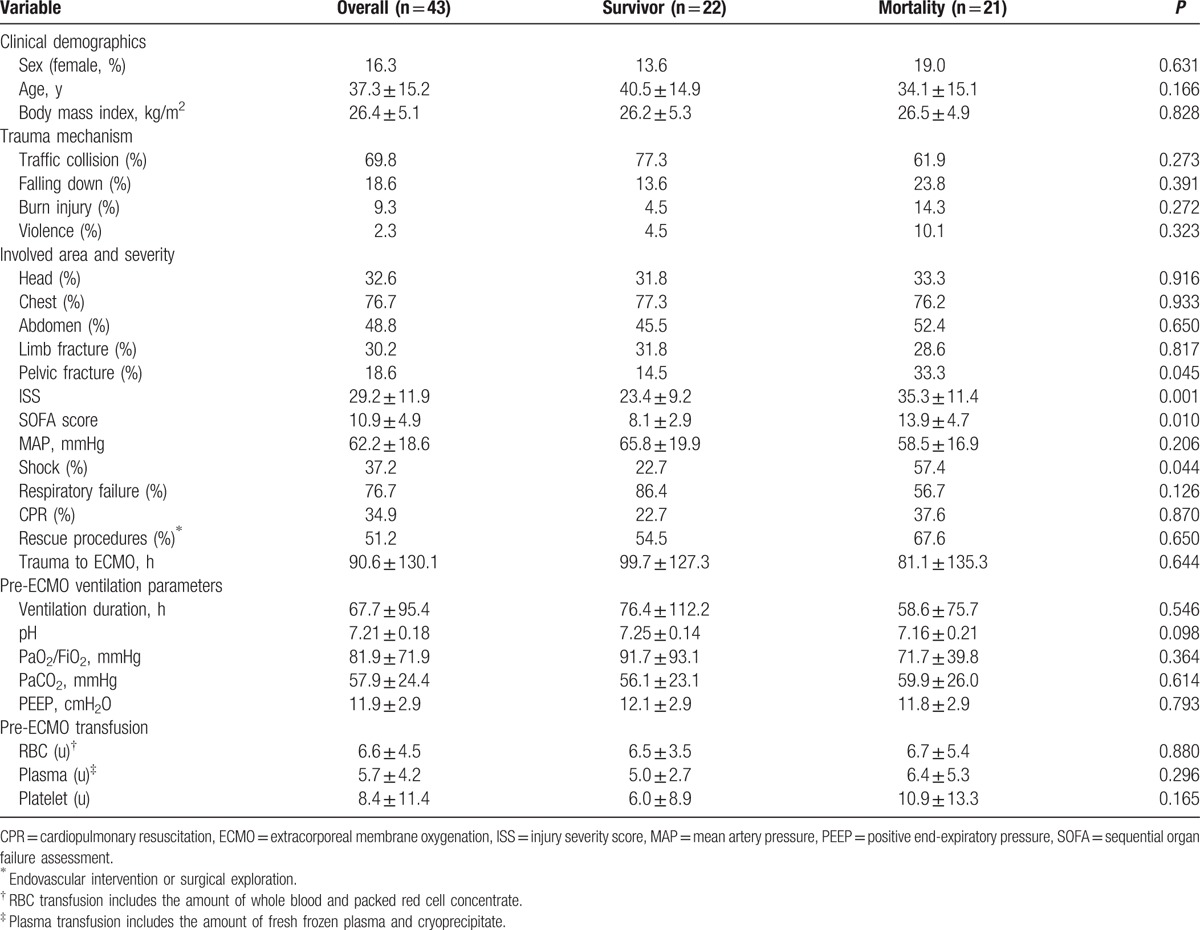
Clinical demographics, trauma mechanisms, involved area, severity, and ventilation parameters for the survivor and mortality groups.

### 3.2 ECMO support and final outcome

Table 2 provides detailed information regarding ECMO support and final outcome. A total of 39.5% of patients underwent the VA mode of support and 37.2% did not initially receive heparin because of bleeding concerns. Although the ECMO flow and mean arterial pressure were similar among survivor and mortality groups, the serum lactate level 8 hours after ECMO support was significantly higher in the mortality group compared to that for the survivor group (74.7 ± 38.7 vs. 40.6 ± 43.7, *P* = 0.043). In addition, 26 patients (60.5%) were weaned off of ECMO support and 22 patients (51.6%) survived to discharge. The mean duration of ECMO support was 162.9 ± 182.7 hours, and the mean length of hospital stay was 41.8 ± 42.5 days. Among 17 patients with VA ECMO support, the average SAVE score is 2.9 ± 3.4.^[16]^ The estimated survival is 42%, which is close to the observed survival in the present study (7/17; 41.2%). The mortality group had a significantly higher rate of RRT (22.7% versus 71.4%, *P* = 0.001) and interventional procedures during ECMO support (18.2% versus 57.1%, *P* = 0.008) compared to those for the survivor group. A total of 34.9% of patients experienced bleeding complications, 14.0% of cerebral infarction, and 7.0% of intracranial hemorrhage events.

**Table 2:**
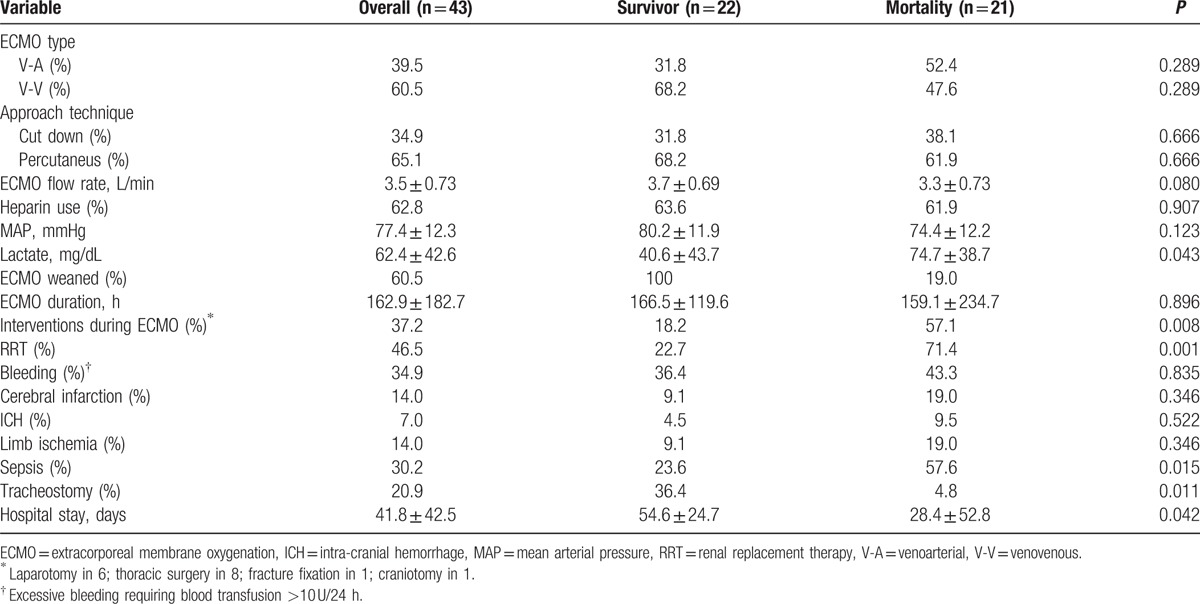
ECMO details and final outcomes for the survivor and mortality groups.

### 3.3 Prognostic factors associated with in-hospital mortality

Table 3 provides the results for the regression analyses, including those for an ISS >30, SOFA score >11.5, serum lactate level >41.8 mg/dL, shock status before ECMO, pelvic fracture, undergoing rescue interventional procedures during ECMO support, and requirement of RRT. Two significant prognostic factors for in-hospital mortality were identified: ISS >30 (odds ratio [OR)], 9.48; 95% confidence interval [CI], 1.04–18.47; *P* = 0.042) and RRT (OR, 8.64; 95% CI, 1.73–26.09; *P* = 0.020). For ISS, the established model revealed a good calibration (Hosmer-Lemeshow test, *P* = 0.71) and discrimination (AUROC 0.763, *P* = 0.04).^[18]^

**Table 3:**
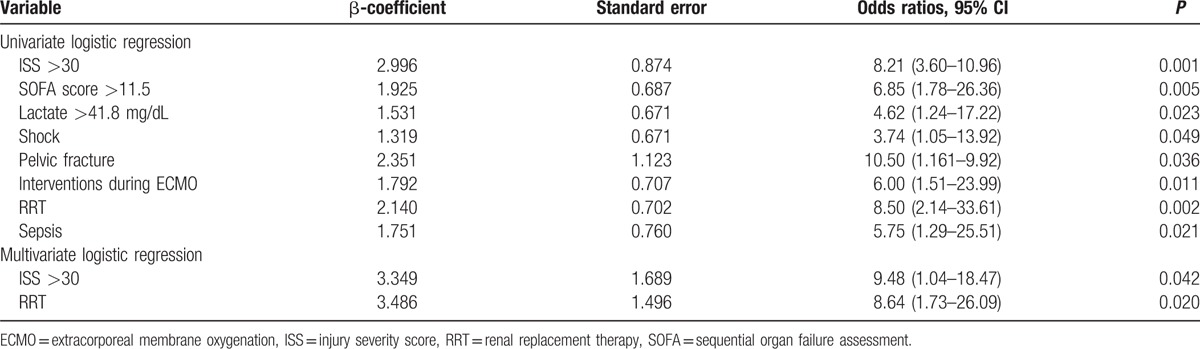
Risk factors for hospital mortality.

### 3.4 Cumulative 1-year survival

**Figure 1:**
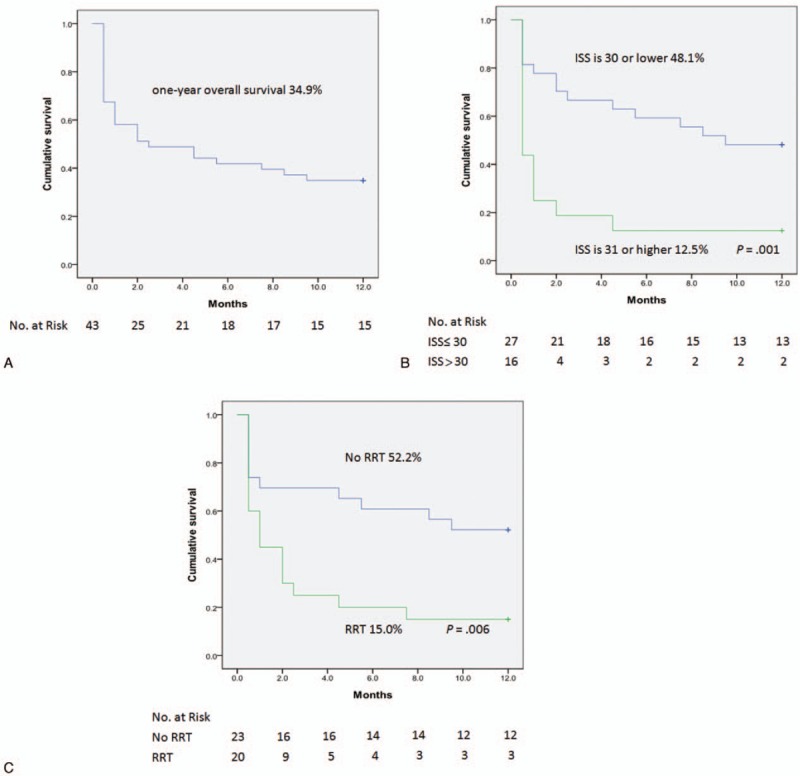
(A) One-year overall cumulative survival rate for 43 patients; (B) survival stratified by the ISS; and (C) survival stratified by the requirement of RRT. ISS = injury severity score, RRT = renal replacement therapy.

**Figure 2:**
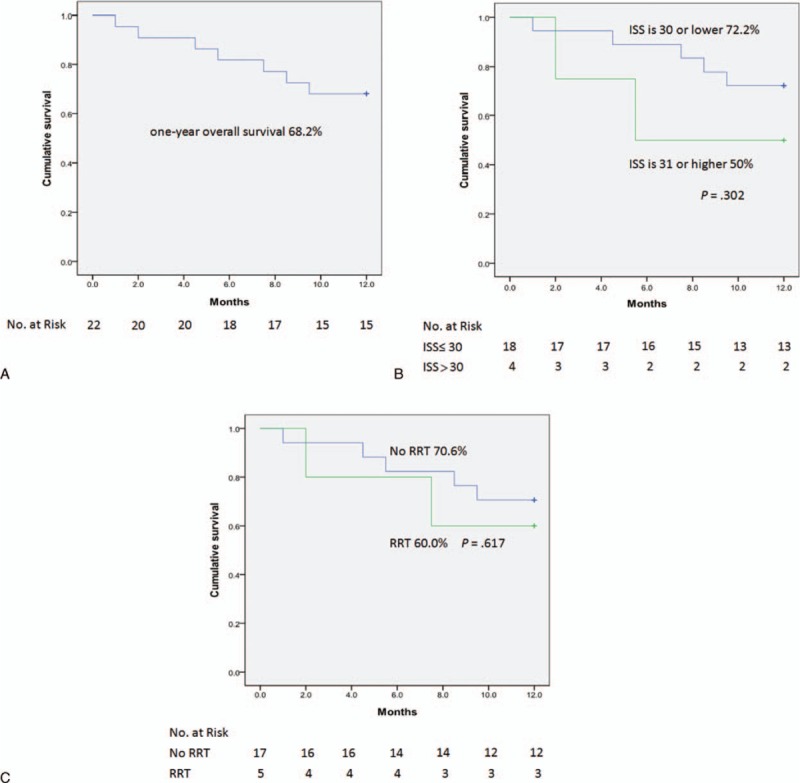
(A) One-year overall cumulative survival rate for 22 patients (excluding those with in-hospital mortality); (B) survival stratified by the ISS; and (C) survival stratified by the requirement of RRT. ISS = injury severity score, RRT = renal replacement therapy.

The overall 1-year cumulative survival rate was 34.9% (Fig. 1A). Moreover, the 1-year survival curves were significantly lower among patients with an ISS >30 compared to those with an ISS ≤30 (12.5% vs. 48.1%, *P* = 0.001; Fig. 1B), and among patients requiring post-ECMO RRT compared to those who did not require post-ECMO RRT (15.0% vs. 52.2%, *P* = 0.006; Fig. 1C). However, when patients with in-hospital mortality were excluded, the 1-year cumulative survival rate was 68.2%, and survival was no longer significantly differed among ISS or post-ECMO RRT groups (Fig. 2).

### 3.5 Cerebral performance

**Figure 3:**
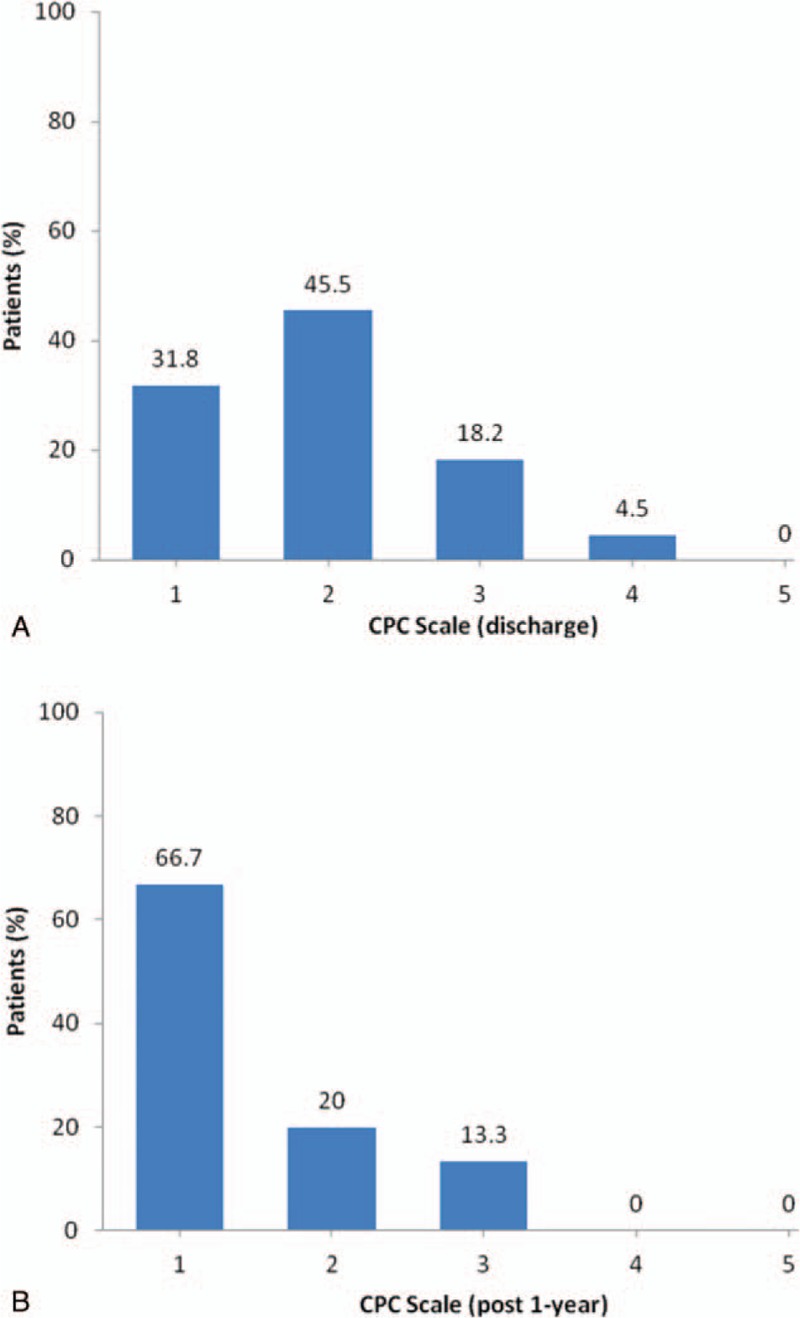
(A) CPC scale for 22 patients survived to discharge; (B) CPC scale for 15 patients with 1-year survival. CPC = cerebral performance categories.

The distribution of each CPC scales is illustrated in Figure 3. When patients discharged, their CPC scales are most centralized on CPC 2 and followed by CPC 1 (45.5% and 31.8%; Fig. 3A). At 1 year after discharge, the prevalence of CPC 1 increased to 66.7%, and CPC 2 reduced to 20% (Fig. 3B).

## 4 Discussion

Multiple traumas associated with cardiopulmonary failure are lethal. Serious bleeding owing to organ destruction or an acute lung injury following a massive blood transfusion may greatly compromise cardiopulmonary function. The use of ECMO provides circulatory and respiratory support, effectively stabilizing unstable hemodynamics and providing a bridge to rescue therapy. In the present study, we reviewed 43 adult patients with post-traumatic cardiopulmonary failure who underwent ECMO. About 60% of patients were able to be weaned off the ECMO support, and about 50% survived to discharge. In addition, a greater trauma severity and the requirement of RRT were found to have an increased risk for in-hospital mortality and inferior mid-term survival.

### 4.1 Timing of ECMO support

Rescue of traumatic cardiopulmonary failure with ECMO remains controversial, mainly because of the associated injuries and bleeding concerns. ECMO support enables optimized tissue oxygenation, stabilizes hemodynamics, and interrupts the vicious cycle of systemic hypoperfusion.^[7,19–21]^ Moreover, patients can be temporarily stabilized and bridged to further rescue interventions. Both in-time evaluation and the initiation of ECMO support require a coordinated approach among trauma specialists and cardiovascular surgeons. In the present study, the mean time interval from the traumatic insult to ECMO was 90.6 ± 130.1 hours. Although the overall percentage of respiratory failure was 76.7%, only 60.5% could be supported using the VV mode of ECMO. For some patients, pulmonary function was supported too late to reverse course, resulting in progression to cardiac and pulmonary failures. Only a timely intervention can prevent rapid deterioration and thus impact the final outcome.

### 4.2 Risks for mortality: injury score and lactate level

Major trauma is commonly defined using an ISS threshold of 15.^[22]^ As reported by Antonelli et al,^[23]^ the SOFA score can reliably describe organ dysfunction/failure in trauma patients, and can be helpful in identifying patients at major risk for a prolonged ICU stay or death. Moreover, Manikis et al^[24]^ identified lactate, which is a product of undesirable anaerobic metabolism, as an indicator of the initial resuscitation predicting mortality in trauma patients. In the present study, an ISS >30 was recognized as an independent risk factor for hospital mortality, and both the SOFA score and lactate levels were higher in mortality group compared to that for the survivor group. This finding suggests that the injury scores and lactate level are important indicators of shock status. For patients with high scores, the threshold for ECMO support may need to be reduced.

### 4.3 Risks for mortality: RRT

Acute renal failure requiring RRT was also an independent predictor of in-hospital mortality in the present study as well as our previous studies.^[25]^ As reported in Askenazi et al,^[26]^ acute kidney injury (AKI) occurred in 70% to 85% of patients with ECMO support, and those who required RRT were at high risk for mortality. Kidney perfusion is autoregulated until the mean arterial pressure falls <80 mmHg. In the present study, the MAP measured before ECMO support was far below this lower limit for both groups, but especially for the mortality group (65.8 ± 19.9 vs. 58.5 ± 16.9 mmHg). Similar to the serum lactate level, renal function can be assumed to be an indicator of systemically inadequate perfusion because of its sensitive fluctuation among critically ill patients. Once acute renal failure develops, the patient should be cautiously re-evaluated for hemodynamic targets and cardiopulmonary function perseverance. Moreover, early and aggressive use of ECMO should be considered.

### 4.4 Heparin-free strategy

Achieving hemostasis in patients with multiple traumas associated with hemorrhagic shock is the primary goal of the initial resuscitation. Application of ECMO in this scenario always raises concern because it may exaggerate coagulopathy, owing to the consumption of coagulation factors and activation of the inflammatory system. A heparin-minimized or heparin-free strategy during the initial ECMO deployment has been evaluated in several studies on patients with post-traumatic shock.^[8,9,27]^ In the present study, a tailored anticoagulation protocol was conducted and 37.2% of patients received rescue surgery or interventional procedures after ECMO support with acceptable bleeding or embolic morbidities. Without hesitation, traumatized patients with cardiopulmonary failure should be considered for ECMO therapy, regardless of bleeding concerns, and the status of “still on ECMO support” should not delay any additional operations if indicated.

### 4.5 One-year outcome

Both an ISS >30 and the requirement of RRT contributed to in-hospital and 1-year mortality. However, with the exclusion of patients with in-hospital mortality, these factors were no longer risk factors for mortality after discharge. Similar result has been described in the study from Chang et al.^[28]^ Patients suffering from extreme injuries, with a high severity score and requiring RRT, were expected to have an early mortality. However, once such patients are stabilized by ECMO and survive to discharge, the mid-term outcome can be promising. Because traumatic insult usually occurs in a normal population, quick and prompt ECMO can restore the cardiopulmonary function to normal status more often than that for non-traumatic populations.

### 4.6 Limitations of this study

Despite the promising results of the present study, several important limitations must be considered. First, the study used a retrospective and nonrandomized control design with very small sample size; bias might exist influencing the homogeneity of the mortality and survivor groups. Second, the decision regarding ECMO consultations was made by individual physicians, without consensus or protocol agreement. A different threshold for ECMO support and ventilator strategies might have also affected the final outcomes. Finally, as a retrospective study, some hemodynamic data, laboratory profiles, and inotropic medication dosage information were not completely analyzed because of missing or incomplete records. This hindered more detailed analyses of physiological fluctuations during ECMO support.

## 5 Conclusions

By providing adequate tissue oxygenation and stabilizing the hemodynamics, ECMO support in selected traumatized patients with cardiopulmonary failure can achieve satisfactory outcomes. Though this is a very small sample size study, multiple organ involvement was common in these scenarios and deterioration easily occurred with shock-related malperfusion. The early deployment of ECMO (before irreversible organ damage), judicious heparin titration therapy, and aggressive correction of associated injuries are key to the final success.
